# Supplementary material for: Comparison of Physicochemical, Microbiological Properties and Bioactive Compounds Content of Grassland Honey and other Floral Origin Honeys
Source: Molecules. 2019 Aug 13;24(16):2932. doi: 10.3390/molecules24162932 (PMC6721125; doi:10.3390/molecules24162932)
Supplement: Supplementary file 1 [file molecules-24-02932-s001.pdf]

Results

Table S1. Results of microbiological determination of honey samples

Honey sample	Microorganism count (cfu/g)										
	SPC	Bacillus spp.	TC	Yeasts	Molds	Honey sample	SPC	Bacillus spp.	TC	Yeasts	Molds
R1	40		-	20	<10	*L9*	20	-	-	10	<10
R2	30	-	-	10	<10	*L10*	<10	-	-	<10	<10
R3	<10	-	-	<10	<10	*L11*	<10	-	-	<10	<10
R4	<10	-	-	<10	<10	*L12*	<10	-	-	<10	<10
R5	<10	-	-	<10	<10	*L13*	20	-	-	10	<10
R6	<10	-	-	<10	<10	*L14*	<10	-	-	<10	<10
R7	<10	-	-	<10	<10	*L15*	10	-	-	<10	<10
R8	<10	-	-	<10	-	*L16*	<10	-	-	<10	<10
R9	<10	-	-	<10	-	*L17*	<10	-	-	<10	<10
R10	<10	-	-	<10	-	*L18*	10	-	-	<10	<10
R11	40	-	-	20	<10	*L19*	<10	-	-	<10	<10
R12	<10	-	-	<10	<10	*L20*	<10	-	-	<10	<10
R13	<10	-	-	<10	-	*L21*	<10	-	-	<10	<10
R14	<10	-	-	<10	-	*A1*	<10	-	-	<10	<10
H1	<10	-	-	<10	<10	*A2*	20	-	-	10	<10
H2	<10	-	-	<10	<10	*A3*	<10	-	-	<10	<10
H3	<10	-	-	<10	<10	*A4*	<10	-	-	<10	<10
H4	<10	-	-	<10	<10	*A5*	<10	-	-	<10	10
H5	10	-	-	<10	<10	*A6*	<10	-	-	<10	<10
H6	<10	-	-	<10	<10	*A7*	<10	-	-	<10	<10
H7	20	-	-	10	<10	*A8*	<10	-	-	<10	10
H8	20	-	-	10	<10	*A9*	10	-	-	<10	<10
H9	<10	-	-	<10	<10	*A10*	<10	-	-	<10	-
P1	<10	-	-	<10	<10	*A11*	<10	-	-	<10	-
P2	<10	-	-	<10	<10	*A12*	20	-	-	10	-
P3	<10	-	-	<10	<10	*A13*	<10	-	-	<10	-
P4	10	-	-	10	<10	*A14*	<10	-	-	<10	<10
P5	<10	-	-	<10	<10	*A15*	<10	-	-	20	<10
P6	20	-	-	<10	10	*A16*	<10	-	-	<10	<10
P7	<10	-	-	<10	<10	*A17*	<10	-	-	<10	-
P8	<10	-	-	<10	<10	*A18*	<10	-	-	<10	<10
P9	30	-	-	20	<10	*A19*	<10	-	-	<10	-
P10	<10	-	-	<10	<10	*A20*	<10	-	-	<10	<10
P11	<10	-	-	<10	<10	*A21*	<10	-	-	<10	<10
P12	<10	-	-	10	<10	*G1*	<10	-	-	<10	<10
P13	<10	-	-	<10	10	*G2*	<10	-	-	<10	-

P14	<10	-	-	<10	<10	*G3*	-	-	-	-	-
P15	<10	-	-	<10	<10	*G4*	-	-	-	-	-
P16	<10	-	-	<10	<10	*G5*	<10	-	-	<10	<10
L1	<10	-	-	-	<10	*G6*	<10	-	-	<10	-
L2	<10	-	-	<10	10	*G7*	<10	-	-	-	-
L3	<10	-	-	<10	<10	*G8*	<10	-	-	<10	-
L4	30	-	-	20	<10	*G9*	<10	-	-	<10	-
L5	<10	-	-	-	<10	*G10*	<10	-	-	<10	-
L6	<10	-	-	<10	<10	*G11*	<10	-	-	<10	-
L7	<10	-	-	<10	<10	*G12*	-	-	-	-	-
L8	<10	-	-	<10	<10						

*R- rape honey; H- honeydew; L- linden honey; P- polyfloral honey; A -acacia honey; G-grassland honey (- absent).

Table S2: Physicochemical parameters of different type of honey from Romania

Sam ple no.	Water content, %	L*	a*	b*	Chroma	H angle, degrees	Con duc tivity, mS/cm	Visco sity, Pa*s	*Sam ple no.*	Water content, %	L*	a*	b*	Chroma	H angle, degrees	Con duc tivity, mS/cm	Vis co sity, Pa*s
R1	17.20	21.67	-0.31	6.23	3.05	87.09	0.11	11.65	***L9***	17.10	31.7	-0.27	14.31	4.30	88.92	0.61	6.24
R2	16.90	25.87	-0.14	7.45	3.10	88.81	0.16	13.24	***L10***	16.50	33.22	-0.13	13.42	4.02	89.45	0.57	7.46
R3	16.70	19.06	-0.38	6.17	3.10	86.51	0.12	12.28	***L11***	17.00	33.49	-0.56	12.89	4.34	87.51	0.58	7.01
R4	17.30	22.18	-0.56	4.98	2.98	83.65	0.16	14.73	***L12***	17.20	35.27	-0.48	14.38	4.48	88.09	0.61	5.17
R5	16.90	38.07	-1.69	15.69	5.26	83.65	0.15	11.82	***L13***	16.80	34.83	-0.31	14.29	4.34	88.76	0.60	8.06
R6	17.80	30.65	-1.27	5.37	3.44	76.77	0.17	10.96	***L14***	17.10	32.71	-0.92	13.26	4.60	86.03	0.61	7.62
R7	17.80	27.41	-0.93	7.21	3.65	82.50	0.12	12.63	***L15***	16.00	31.65	-0.84	14.67	4.75	86.72	0.55	4.89
R8	17.90	24.85	-0.20	5.63	2.82	88.23	0.15	12.41	***L16***	17.80	35.23	-0.38	13.81	4.33	88.43	0.63	5.83
R9	18.00	25.14	-0.28	4.56	2.66	86.51	0.19	11.98	***L17***	16.50	33.45	-0.21	13.45	4.13	89.11	0.59	7.22
R10	16.90	22.98	-0.51	7.85	3.52	86.51	0.11	13.85	***L18***	17.90	34.92	-1.21	13.98	4.84	85.05	0.69	6.68
R11	17.50	26.72	-0.16	6.22	2.89	88.81	0.13	11.47	***L19***	17.00	31.76	-1.04	12.43	4.55	85.22	0.62	5.28
R12	17.20	17.61	-1.25	8.17	3.98	81.36	0.16	13.72	***L20***	17.40	33.16	-0.42	13.97	4.39	88.28	0.64	7.16
R13	17.70	29.02	-0.34	5.54	2.94	86.51	0.14	12.74	***L21***	16.60	32.89	-0.25	14.34	4.29	89.00	0.58	4.76
R14	16.80	33.68	-2.19	18.61	5.79	83.08	0.11	14.29	***A1***	17.10	45.28	-0.76	16.25	4.90	87.32	0.23	3.67
H1	15.90	19.67	9.29	8.35	5.94	41.82	0.58	9.47	***A2***	17.80	47.87	-1.16	14.62	4.90	85.46	0.32	3.98
H2	16.20	21.41	7.62	6.92	5.39	42.40	0.65	7.65	***A3***	17.30	49.12	-1.39	14.98	5.05	84.70	0.25	2.14
H3	17.40	21.14	5.98	7.13	5.12	49.84	0.69	8.71	***A4***	16.90	50.38	-0.45	16.38	4.72	88.43	0.22	4.08
H4	17.90	23.72	6.52	10.17	5.74	57.29	0.73	8.92	***A5***	17.10	46.12	-1.54	14.92	5.10	84.11	0.28	3.15
H5	16.90	21.56	7.23	9.67	5.80	53.28	0.66	7.14	***A6***	16.90	48.18	-0.83	13.28	4.56	86.43	0.24	3.78
H6	16.50	21.08	7.14	10.24	5.87	55.00	0.59	9.12	***A7***	16.70	45.96	-1.26	14.82	4.97	85.14	0.27	4.14
H7	16.80	22.39	6.28	8.37	5.40	53.28	0.64	8.61	***A8***	17.50	46.24	-0.83	16.34	4.95	87.09	0.30	2.54
H8	15.70	23.84	5.35	6.17	4.80	49.27	0.57	8.56	***A9***	16.70	48.22	-1.69	14.18	5.07	83.20	0.26	4.27
H9	16.30	20.59	8.16	8.22	5.72	45.26	0.61	7.98	***A10***	16.50	52.39	-1.34	15.12	5.05	84.94	0.22	3.29
P1	17.50	36.82	5.16	13.56	5.95	69.17	0.40	6.25	***A11***	18.00	50.16	-0.98	15.69	4.95	86.43	0.35	3.62
P2	17.90	35.15	3.99	13.98	5.74	74.07	0.42	8.21	***A12***	17.60	51.49	-1.29	14.89	4.99	85.05	0.33	2.96
P3	16.20	37.24	3.89	12.74	5.54	73.02	0.35	4.18	***A13***	17.20	48.64	-0.73	16.27	4.89	87.43	0.30	3.46
P4	17.30	33.19	4.23	13.99	5.80	73.18	0.42	6.82	***A14***	16.80	47.29	-0.89	15.56	4.89	86.73	0.28	3.89
P5	16.40	35.67	4.78	14.82	6.04	72.12	0.38	7.18	***A15***	17.50	50.62	-1.19	13.25	4.73	84.87	0.31	2.12
P6	15.90	36.73	4.45	13.25	5.75	71.44	0.32	4.98	***A16***	17.20	51.27	-1.27	16.38	5.17	85.57	0.29	2.56

P7	16.80	36.09	5.48	13.65	6.04	68.13	0.41	8.28	***A17***	16.40	51.73	-1.08	13.95	4.77	85.57	0.25	3.25
P8	16.60	35.04	5.35	14.28	6.09	69.46	0.39	6.87	***A18***	16.90	52.61	-1.13	13.25	4.70	85.13	0.28	4.07
P9	17.30	38.73	5.67	14.63	6.21	68.82	0.44	5.36	***A19***	17.60	51.37	-1.56	16.29	5.29	84.53	0.31	2.86
P10	16.90	37.81	3.24	13.38	5.46	76.39	0.41	8.62	***A20***	16.30	49.28	-1.27	14.37	4.92	84.95	0.27	3.52
P11	16.10	38.08	5.98	13.61	6.13	66.28	0.28	5.84	***A21***	17.80	50.15	-0.72	15.28	4.76	87.30	0.33	2.92
P12	17.70	32.83	5.12	14.28	6.04	70.28	0.45	6.50	***G1***	17.60	34.56	2.11	12.59	5.00	80.49	0.28	8.64
P13	17.10	35.61	4.06	12.94	5.61	72.58	0.42	5.12	***G2***	17.20	29.34	6.29	12.15	5.99	62.63	0.35	6.39
P14	15.90	39.65	4.56	13.99	5.88	71.95	0.35	7.08	***G3***	16.30	29.66	1.07	13.16	4.66	85.35	0.29	7.12
P15	16.80	31.90	4.78	13.82	5.90	70.92	0.38	6.62	***G4***	16.70	32.14	3.89	14.09	5.73	74.57	0.33	8.05
P16	16.10	37.56	4.28	13.46	5.74	72.36	0.33	8.12	***G5***	16.90	34.63	0.31	14.59	4.38	88.78	0.34	8.29
L1	16.90	31.97	-0.36	14.94	4.47	88.62	0.62	5.38	***G6***	17.60	32.71	0.92	13.51	4.63	86.11	0.24	7.76
L2	17.90	27.92	-0.78	13.72	4.59	86.75	0.72	6.15	***G7***	17.30	31.75	1.84	13.25	5.00	82.10	0.28	8.17
L3	16.70	35.14	-0.12	14.41	4.14	89.52	0.60	7.04	***G8***	16.90	35.23	0.38	14.37	4.41	88.49	0.31	8.54
L4	16.90	33.29	-0.28	14.19	4.30	88.87	0.61	6.21	***G9***	17.10	33.35	2.21	12.23	4.98	79.76	0.34	7.23
L5	16.30	32.68	-0.34	13.49	4.26	88.56	0.59	5.78	***G10***	16.90	34.92	1.21	13.06	4.71	84.71	0.28	8.2
L6	17.60	35.07	-0.35	12.59	4.14	88.41	0.66	7.37	***G11***	16.70	31.90	1.04	13.08	4.64	85.46	0.32	7.14
L7	17.20	31.18	-0.67	13.56	4.50	87.17	0.63	5.92	***G12***	17.10	33.06	0.42	13.67	4.35	88.24	0.35	6.99
L8	16.90	32.63	-0.34	14.72	4.42	88.68	0.59	6.54									

Table S3: Sugars content of different type of honey from the North of Romania

	Glucose,g/100g	Fructose, g/100g	Sucrose,g/100g	Treha lose,g/100g	Melezitose,g/100g	Maltose,g/100g	Erlose,g/100g	Turanose,g/100g	Raffinose,g/100g	G/F	G/W
**R1**	42.55	28.58	1.60	0.00	0.00	0.26	0.00	0.12	0.00	1.48	2.47
**R2**	42.04	28.74	0.00	0.00	0.00	0.32	0.00	0.12	0.01	1.46	2.48
**R3**	41.70	29.12	0.01	0.01	0.00	0.93	0.00	0.10	0.00	1.43	2.49
**R4**	41.58	29.14	0.00	0.01	0.00	0.33	0.00	0.34	0.20	1.42	2.40
**R5**	40.73	29.73	0.32	0.01	0.01	0.06	0.10	0.45	0.00	1.36	2.41
**R6**	40.42	30.01	1.59	0.01	0.01	0.00	0.00	0.31	0.02	1.34	2.27
**R7**	39.79	30.24	1.45	0.01	0.01	0.00	0.00	0.28	0.01	1.31	2.23
**R8**	39.37	30.50	0.97	0.01	0.01	0.00	0.00	0.12	0.01	1.29	2.20
**R9**	39.13	30.87	1.77	0.02	0.02	1.03	0.00	0.05	0.43	1.26	2.17
**R10**	38.86	31.01	0.93	0.02	0.02	1.25	0.00	0.02	0.01	1.25	2.30
**R11**	38.52	31.15	1.49	0.02	0.02	0.00	0.00	0.06	0.02	1.23	2.20
**R12**	38.35	31.52	0.79	0.02	0.02	0.00	0.00	0.07	0.02	1.21	2.23
**R13**	38.25	31.54	0.57	0.03	0.02	0.00	0.00	0.30	0.00	1.21	2.16
**R14**	38.02	31.54	1.63	0.03	0.02	0.00	0.00	0.05	0.01	1.20	2.26
**H1**	37.21	32.14	0.74	0.03	4.18	0.00	0.04	0.06	0.02	1.15	2.34
**H2**	37.01	32.20	1.80	0.04	1.24	0.00	0.00	0.07	0.02	1.14	2.28
**H3**	36.92	32.28	1.36	0.04	4.47	0.00	0.49	0.20	0.01	1.14	2.12
**H4**	36.42	32.32	1.41	0.04	4.75	0.00	1.54	0.12	0.01	1.12	2.03
**H5**	36.39	32.43	1.31	0.04	5.56	0.00	0.75	0.16	0.00	1.12	2.15
**H6**	36.20	32.45	1.52	0.04	5.76	0.76	0.00	0.13	0.02	1.11	2.19
**H7**	36.16	32.60	1.34	0.04	5.77	0.50	0.18	0.09	0.01	1.10	2.15
**H8**	35.72	32.62	1.45	0.04	5.78	0.52	0.00	0.07	0.00	1.09	2.27
**H9**	35.68	32.68	1.32	0.04	5.84	0.69	0.79	0.03	0.00	1.09	2.18
**P1**	32.31	37.54	0.71	0.23	0.58	0.05	0.00	0.17	0.01	0.86	1.84
**P2**	32.31	37.63	1.08	0.23	0.58	0.00	8.85	0.00	0.02	0.85	1.80
**P3**	32.30	37.68	0.58	0.23	0.61	0.00	0.24	0.23	0.05	0.85	1.99
**P4**	32.18	38.03	1.07	0.24	0.61	0.00	0.00	0.22	0.04	0.84	1.86
**P5**	32.18	38.13	0.19	0.24	0.62	0.00	3.23	0.07	0.04	0.84	1.96
**P6**	32.13	38.27	0.56	0.24	0.63	0.00	0.03	0.44	0.01	0.83	2.02
**P7**	32.08	38.41	1.83	0.24	0.64	0.00	0.02	0.08	0.02	0.83	1.91
**P8**	31.96	38.71	1.95	0.24	0.66	0.00	0.18	0.16	0.02	0.82	1.92
**P9**	31.95	38.74	1.94	0.24	0.68	0.00	0.18	0.64	0.05	0.82	1.84
**P10**	31.91	38.82	1.47	0.24	0.69	0.00	0.51	0.10	0.03	0.82	1.88
**P11**	31.81	38.86	1.50	0.26	0.72	0.00	0.00	0.19	0.01	0.81	1.97

**P12**	31.71	39.00	1.40	0.26	0.72	0.00	6.39	0.71	0.01	0.81	1.79
**P13**	31.66	39.13	1.12	0.27	0.77	0.00	0.00	0.06	0.02	0.80	1.85
**P14**	31.66	39.19	1.50	0.27	0.77	0.00	0.00	0.21	0.03	0.80	1.99
**P15**	31.65	39.34	0.22	0.28	0.94	0.00	0.00	0.16	0.04	0.80	1.88
**P16**	31.62	39.35	0.75	0.28	1.12	0.08	0.00	0.23	0.03	0.80	1.96
**L1**	31.56	39.43	1.45	0.28	1.13	0.06	0.00	0.18	0.04	0.80	1.86
**L2**	31.56	39.45	0.29	0.28	1.30	0.01	0.00	0.02	0.00	0.80	1.76
**L3**	31.52	39.55	0.15	0.29	1.36	0.02	0.07	0.22	0.38	0.79	1.88
**L4**	31.47	39.83	1.22	0.29	1.50	0.01	0.01	0.02	0.03	0.79	1.86
**L5**	31.43	40.20	0.30	0.30	1.62	0.23	0.00	0.24	0.19	0.78	1.92
**L6**	31.08	40.28	1.67	0.30	1.73	2.34	0.68	0.00	0.00	0.77	1.76
**L7**	30.93	40.41	0.29	0.31	1.74	0.00	0.07	0.19	0.09	0.76	1.79
**L8**	30.90	40.50	0.84	0.31	1.83	0.00	0.07	0.04	0.04	0.76	1.82
**L9**	30.89	40.51	0.31	0.31	1.94	0.00	0.02	0.04	0.00	0.76	1.80
**L10**	30.89	40.56	0.22	0.32	2.01	0.00	0.00	0.03	0.00	0.76	1.87
**L11**	30.83	40.57	0.66	0.32	2.09	0.00	0.17	0.03	0.00	0.76	1.81
**L12**	30.81	40.63	0.51	0.33	2.09	0.00	0.04	0.00	0.02	0.75	1.79
**L13**	30.74	40.69	0.16	0.33	2.10	0.00	0.33	0.02	0.03	0.75	1.83
**L14**	30.71	40.70	0.08	0.34	2.18	0.00	0.00	0.02	0.01	0.75	1.79
**L15**	30.64	40.71	0.24	0.34	2.33	0.00	0.01	0.40	0.03	0.75	1.91
**L16**	30.64	40.91	0.16	0.34	2.34	0.17	0.01	0.01	0.04	0.74	1.72
**L17**	30.43	40.97	0.64	0.35	2.35	0.23	0.04	0.08	0.01	0.74	1.84
**L18**	30.43	41.00	0.24	0.35	2.38	0.93	0.02	0.11	0.01	0.74	1.70
**L19**	30.42	41.01	0.54	0.36	2.43	0.06	0.00	0.18	0.01	0.74	1.78
**L20**	30.30	41.10	0.39	0.37	2.55	0.01	0.03	0.11	0.04	0.73	1.74
**L21**	30.39	41.15	0.37	0.38	2.56	0.01	0.01	0.36	0.00	0.73	1.83

	Glucose,g/100g	Fructose, g/100g	Sucrose,g/100g	Trehalose,g/100g	Melezitose,g/100g	Maltose,g/100g	Erlose,g/100g	Turanose,g/100g	Raffinose,g/100g	G/F	G/M
**A1**	28.26	42.65	0.05	0.83	0.03	0.00	0.04	0.01	0.01	0.66	1.65
**A2**	28.25	42.84	0.32	0.85	0.03	0.00	0.00	0.18	0.00	0.66	1.58
**A3**	28.20	43.00	0.51	0.87	0.04	0.00	0.03	0.06	0.01	0.65	1.63
**A4**	28.11	42.99	0.23	0.87	0.04	0.00	0.04	0.17	0.02	0.65	1.66
**A5**	27.89	43.01	0.06	0.93	0.04	0.00	0.99	0.15	0.01	0.64	1.63
**A6**	27.57	43.57	0.44	0.99	0.04	0.00	2.50	0.45	0.02	0.63	1.63
**A7**	27.27	43.65	0.04	1.10	0.04	0.00	0.41	0.03	0.02	0.62	1.63
**A8**	27.17	43.65	1.16	1.26	0.04	0.01	0.04	0.36	0.11	0.62	1.55
**A9**	26.81	43.66	1.56	1.35	0.04	0.01	0.07	0.21	0.01	0.61	1.60
**A10**	26.73	43.67	0.38	1.41	0.03	0.26	0.03	0.00	0.02	0.61	1.62
**A11**	26.69	43.77	1.43	1.65	0.05	0.01	0.00	0.00	0.03	0.61	1.48
**A12**	26.50	43.86	1.04	1.72	0.04	0.01	0.00	0.22	0.00	0.60	1.50
**A13**	25.56	44.24	0.99	1.80	0.04	0.05	0.00	0.01	0.00	0.57	1.48
**A14**	25.33	44.38	0.83	2.23	0.07	0.09	0.00	0.01	0.00	0.57	1.50
**A15**	25.03	44.46	0.80	2.40	0.05	0.02	0.00	0.11	0.00	0.56	1.43
**A16**	24.86	44.68	0.29	2.70	0.05	0.02	0.00	0.37	0.00	0.55	1.44
**A17**	24.75	45.22	1.58	2.75	0.04	0.02	4.28	0.04	0.02	0.54	1.51
**A18**	24.28	45.58	0.84	2.83	0.05	0.02	0.02	0.00	0.13	0.53	1.43
**A19**	23.96	45.60	0.07	3.17	0.06	0.02	0.00	0.00	0.05	0.52	1.36
**A20**	23.63	45.83	0.29	3.48	0.07	0.62	0.03	0.18	0.02	0.51	1.45
**A21**	23.63	45.98	0.30	3.74	0.04	0.00	2.58	0.01	0.01	0.51	1.32
**G1**	37.96	31.85	1.96	0.03	0.03	0.00	0.52	0.01	0.01	1.19	2.15
**G2**	37.53	32.04	0.99	0.03	0.03	0.00	0.00	0.32	0.00	1.17	2.18
**G3**	37.42	32.06	0.95	0.03	0.08	0.00	0.00	0.16	0.00	1.16	2.29
**G4**	37.22	32.08	1.73	0.03	0.03	0.00	0.07	0.44	0.03	1.16	2.22
**G5**	34.26	41.22	0.67	0.38	2.58	0.78	0.07	0.11	0.00	0.83	2.02
**G6**	35.09	41.23	0.27	0.38	2.66	0.87	0.07	0.14	0.00	0.85	1.99
**G7**	38.09	37.29	0.37	0.39	2.78	0.01	0.07	0.01	0.02	1.02	2.20
**G8**	39.05	37.42	0.31	0.40	2.79	0.01	0.01	0.09	0.03	1.04	2.31
**G9**	39.98	37.54	0.64	0.43	2.79	0.10	0.01	0.38	0.03	1.06	2.33

**G10**	34.49	38.58	0.30	0.47	2.82	0.06	0.02	0.43	0.03	0.89	2.04
**G11**	34.36	39.69	0.68	0.47	2.86	0.33	0.00	0.11	0.04	0.86	2.05
**G12**	36.34	38.71	0.33	0.47	3.11	0.06	0.02	0.11	0.01	0.94	2.12

Table S4 The results of the concentration of the twelve phenolic compounds

	Gallic acid, mg/100g	Protocatechuic acid,mg g/100g	4- hydroxybenzoic acid, mg/100g	Vanilic acid, mg/100g	Chlorogenic acid. mg/100g	Caffeic acid, mg/100g	P-coumaric acid, mg/100g	Rosmarinic acid, mg/100g	Myricetin, mg/100g	Quercitin, mg/100g	Luteolin, mg/100g	Kaempferol, mg/100g	Total poliphenols, mg/100g	Total phenolic content Folin Ciocalteu, mg GAE/100g
**R1**	0.00	0.00	0.00	0.00	0.00	0.04	0.04	0.00	0.00	0.00	0.00	0.00	0.08	0.12
**R2**	0.00	0.00	0.00	0.00	0.00	0.09	0.00	0.00	0.00	0.00	0.00	0.00	0.09	0.12
**R3**	0.00	0.00	0.00	0.00	0.00	0.05	0.03	0.00	0.00	0.00	0.00	0.00	0.09	0.12
**R4**	0.00	0.00	0.00	0.00	0.00	0.00	0.12	0.00	0.00	0.00	0.00	0.00	0.12	0.15
**R5**	0.00	0.00	0.00	0.14	0.00	0.00	0.00	0.00	0.00	0.00	0.00	0.00	0.14	0.17
**R6**	0.00	0.00	0.14	0.00	0.00	0.00	0.00	0.00	0.00	0.00	0.00	0.00	0.14	0.17
**R7**	0.00	0.00	0.00	0.00	0.00	0.15	0.00	0.00	0.00	0.00	0.00	0.00	0.15	0.17
**R8**	0.00	0.00	0.00	0.00	0.00	0.10	0.05	0.00	0.00	0.00	0.00	0.00	0.15	0.17
**R9**	0.00	0.00	0.00	0.00	0.00	0.15	0.00	0.00	0.00	0.00	0.00	0.00	0.15	0.19
**R10**	0.00	0.00	0.00	0.00	0.00	0.00	0.16	0.00	0.00	0.00	0.00	0.00	0.16	0.17
**R11**	0.00	0.00	0.00	0.00	0.17	0.00	0.00	0.00	0.00	0.00	0.00	0.00	0.17	0.17
**R12**	0.00	0.00	0.00	0.00	0.00	0.11	0.07	0.00	0.00	0.00	0.00	0.00	0.18	0.18
**R13**	0.07	0.00	0.00	0.00	0.13	0.00	0.00	0.00	0.00	0.00	0.00	0.00	0.20	0.21
**R14**	0.00	0.21	0.00	0.00	0.00	0.00	0.00	0.00	0.00	0.00	0.00	0.00	0.21	0.22
**H1**	0.02	0.00	0.00	3.15	0.00	0.00	0.23	5.89	2.34	2.67	0.00	0.55	14.87	14.94
**H2**	0.02	0.00	0.00	4.42	0.00	0.37	0.23	5.01	2.11	2.32	0.00	0.41	14.92	14.95
**H3**	0.35	13.32	0.00	0.00	0.10	0.06	0.05	0.18	0.33	0.00	0.71	0.00	15.12	15.16
**H4**	0.00	6.51	0.99	0.00	1.78	0.47	0.11	0.00	6.60	0.00	0.00	0.00	16.48	16.56
**H5**	0.61	2.03	0.65	18.04	1.16	0.07	0.07	0.00	0.00	0.00	0.00	1.18	23.85	23.96
**H6**	0.04	0.08	0.00	1.21	0.25	0.00	0.19	3.28	0.14	21.21	0.00	0.26	26.70	26.78
**H7**	0.00	0.00	0.00	24.81	0.00	5.53	0.00	0.00	0.00	0.00	0.00	0.00	30.34	30.35
**H8**	0.26	0.00	0.00	0.00	0.00	0.20	0.17	51.11	0.00	0.00	0.00	0.00	51.76	51.78
**H9**	0.00	0.00	0.00	0.00	0.00	0.00	0.02	60.60	19.75	0.00	0.00	0.00	80.38	80.47
**P1**	5.22	0.52	0.00	0.00	0.52	0.10	0.15	0.00	0.00	0.00	0.00	0.00	6.54	6.55
**P2**	0.00	0.00	0.00	0.00	0.00	1.84	0.26	4.67	0.00	0.00	0.00	0.00	6.78	6.78
**P3**	0.00	0.06	0.00	0.00	0.00	0.00	0.22	4.66	2.14	0.00	0.00	0.00	7.09	7.15
**P4**	0.02	0.00	0.00	1.30	0.00	0.00	0.23	5.95	0.00	0.00	0.00	0.00	7.51	7.52
**P5**	0.00	0.00	0.00	0.00	0.00	0.00	0.00	0.00	1.93	6.13	0.00	0.00	8.06	8.12
**P6**	5.23	0.00	0.00	0.00	0.00	0.01	2.32	0.53	0.00	0.00	0.00	0.00	8.11	8.12
**P7**	2.27	0.65	0.49	1.91	0.22	0.12	0.00	2.27	0.00	0.50	0.00	0.00	8.46	8.49
**P8**	5.21	0.07	0.52	0.62	1.16	0.07	0.07	0.00	0.00	0.00	0.00	1.21	8.98	9.04
**P9**	0.00	0.04	0.00	2.27	0.00	0.00	0.25	5.45	0.00	0.00	1.12	0.00	9.14	9.16
**P10**	0.01	0.01	0.00	3.10	0.00	0.08	0.18	5.14	0.36	0.00	0.00	0.41	9.32	9.41
**P11**	0.03	0.08	0.00	1.28	0.00	0.00	0.02	4.46	1.09	2.23	0.00	0.16	9.38	9.40
**P12**	0.02	0.00	0.00	1.18	0.00	0.00	0.21	5.46	2.26	0.31	0.00	0.44	9.91	9.94
**P13**	0.00	0.01	0.00	2.41	0.00	0.00	0.21	4.88	0.00	2.63	0.00	0.56	10.72	10.73
**P14**	2.29	0.65	0.28	1.91	0.19	0.09	0.00	5.26	0.00	0.55	0.00	0.00	11.23	11.31
**P15**	0.02	0.08	0.00	0.97	0.00	0.00	0.23	5.52	0.12	4.42	0.00	0.60	11.99	12.36
**P16**	0.00	0.22	0.00	2.25	0.00	0.00	0.25	6.57	0.14	2.86	0.00	0.48	12.81	12.94
**L1**	0.00	0.63	0.00	0.00	0.00	0.28	0.00	0.02	0.00	0.00	0.00	0.00	0.94	0.99

**L2**	0.64	0.29	0.00	0.00	0.00	0.03	0.00	0.00	0.00	0.00	0.00	0.00	0.97	1.06
**L3**	0.14	0.89	0.00	0.00	0.13	0.00	0.00	0.00	0.00	0.00	0.00	0.00	1.18	1.31
**L4**	1.27	0.00	0.00	0.00	0.00	0.00	0.00	0.00	0.00	0.00	0.00	0.00	1.27	1.30
**L5**	1.42	0.00	0.00	0.00	0.00	0.00	0.00	0.00	0.00	0.00	0.00	0.00	1.42	1.44
**L6**	1.66	0.00	0.00	0.00	0.00	0.00	0.00	0.00	0.00	0.00	0.00	0.00	1.66	1.68
**L7**	0.00	0.00	0.00	0.00	0.00	0.03	1.25	0.00	0.41	0.00	0.00	0.00	1.70	1.72
**L8**	0.00	0.00	0.00	0.00	0.10	0.04	0.00	0.00	1.59	0.00	0.00	0.00	1.75	1.78
**L9**	0.32	0.00	0.00	0.00	0.00	1.20	0.26	0.00	0.00	0.00	0.00	0.00	1.78	1.79
**L10**	1.79	0.00	0.00	0.00	0.00	0.00	0.00	0.00	0.00	0.00	0.00	0.00	1.79	1.79
**L11**	0.00	0.00	0.00	0.00	0.00	0.49	0.37	0.00	0.94	0.00	0.00	0.00	1.81	1.90
**L12**	1.06	0.21	0.50	0.00	0.22	0.00	0.00	0.10	0.00	0.00	0.00	0.00	2.10	2.14
**L13**	2.13	0.00	0.00	0.00	0.00	0.00	0.00	0.00	0.00	0.00	0.00	0.00	2.13	2.18
**L14**	0.00	0.00	0.00	0.00	0.17	0.09	0.44	0.00	1.31	0.00	0.00	0.20	2.23	2.26
**L15**	3.96	0.00	0.00	0.00	0.00	0.00	0.00	0.00	0.00	0.00	0.00	0.00	3.96	3.98
**L16**	3.97	0.00	0.00	0.00	0.00	0.00	0.01	0.00	0.00	0.00	0.00	0.00	3.98	4.02
**L17**	0.00	0.00	0.30	0.00	0.00	0.00	0.00	4.67	0.00	0.00	0.00	0.00	4.98	5.04
**L18**	2.33	0.00	0.00	0.00	0.00	0.00	0.00	0.68	1.92	0.06	0.00	0.00	5.01	5.05
**L19**	2.33	0.66	0.16	0.00	0.27	0.04	0.00	1.03	0.32	0.48	0.00	0.00	5.32	5.35
**L20**	0.01	1.72	0.50	0.00	2.15	0.00	0.00	1.02	0.00	0.00	0.00	0.00	5.42	5.49
**L21**	0.00	0.20	0.54	0.00	0.19	0.00	0.10	4.40	0.00	0.00	0.00	0.00	5.44	5.49
**A1**	0.20	0.00	0.00	0.00	0.00	0.00	0.00	0.00	0.00	0.00	0.00	0.00	0.20	0.28
**A2**	0.00	0.00	0.02	0.00	0.00	0.00	0.00	0.00	0.00	0.00	0.00	0.00	0.02	0.04
**A3**	0.00	0.00	0.00	0.00	0.00	0.00	0.29	0.00	0.00	0.00	0.00	0.00	0.28	0.32
**A4**	0.00	0.00	0.00	0.00	0.00	0.04	0.00	0.00	0.00	0.00	0.00	0.00	0.04	0.05
**A5**	0.00	0.00	0.00	0.00	0.00	0.00	0.04	0.00	0.00	0.00	0.00	0.00	0.04	0.07
**A6**	0.00	0.00	0.00	0.00	0.00	0.04	0.00	0.00	0.00	0.00	0.00	0.00	0.04	0.06
**A7**	0.00	0.00	0.00	0.00	0.00	0.05	0.00	0.00	0.00	0.00	0.00	0.00	0.05	0.06
**A8**	0.00	0.00	0.00	0.00	0.00	0.05	0.00	0.00	0.00	0.00	0.00	0.00	0.05	0.07
**A9**	0.00	0.00	0.00	0.00	0.00	0.05	0.00	0.00	0.00	0.00	0.00	0.00	0.05	0.08
**A10**	0.00	0.00	0.00	0.00	0.00	0.05	0.00	0.00	0.00	0.00	0.00	0.00	0.05	0.07
**A11**	0.00	0.00	0.00	0.00	0.06	0.00	0.00	0.00	0.00	0.00	0.00	0.00	0.06	0.08
**A12**	0.06	0.00	0.00	0.00	0.00	0.00	0.00	0.00	0.00	0.00	0.00	0.00	0.06	0.09
**A13**	0.00	0.00	0.00	0.00	0.00	0.06	0.00	0.00	0.00	0.00	0.00	0.00	0.06	0.12
**A14**	0.00	0.00	0.00	0.00	0.00	0.06	0.00	0.00	0.00	0.00	0.00	0.00	0.06	0.09
**A15**	0.00	0.00	0.00	0.00	0.00	0.04	0.03	0.00	0.00	0.00	0.00	0.00	0.07	0.09
**A16**	0.00	0.00	0.00	0.00	0.00	0.06	0.02	0.00	0.00	0.00	0.00	0.00	0.07	0.11
**A17**	0.08	0.00	0.00	0.00	0.00	0.00	0.00	0.00	0.00	0.00	0.00	0.00	0.08	0.10
**A18**	0.00	0.00	0.00	0.00	0.00	0.08	0.00	0.00	0.00	0.00	0.00	0.00	0.08	0.09
**A19**	0.00	0.00	0.00	0.09	0.00	0.00	0.00	0.00	0.00	0.00	0.00	0.00	0.09	0.10
**A20**	0.00	0.00	0.00	0.00	0.00	0.09	0.00	0.00	0.00	0.00	0.00	0.00	0.09	0.11
**A21**	0.00	0.00	0.00	0.00	0.14	0.00	0.00	0.00	0.00	0.00	0.00	0.00	0.14	0.16
**G1**	0.01	0.00	0.00	3.16	0.00	0.05	0.23	5.16	2.67	2.46	0.92	0.00	14.67	14.69
**G2**	0.00	0.16	0.00	12.76	0.00	0.00	0.27	6.02	0.48	4.26	0.00	0.87	24.81	24.82
**G3**	0.00	4.03	4.78	0.00	4.67	1.59	24.23	0.00	0.00	0.00	0.00	0.00	39.30	39.33
**G4**	0.00	0.00	0.00	44.81	0.00	0.00	0.00	1.04	0.00	0.00	0.00	0.00	45.85	45.88
**G5**	0.00	0.05	0.00	3.00	0.00	0.00	0.22	4.94	2.11	2.20	0.00	0.45	12.97	13.01
**G6**	0.04	0.05	0.00	3.10	0.00	0.00	0.24	5.51	2.24	2.32	0.00	0.05	13.54	13.57
**G7**	5.23	8.85	0.00	0.00	0.00	0.00	0.00	0.00	0.00	0.00	0.00	0.00	14.08	14.12
**G8**	0.02	0.01	0.00	7.25	0.00	0.00	0.02	6.02	0.69	0.00	0.00	0.47	14.47	14.53
**G9**	0.02	0.00	0.00	2.55	0.00	0.00	0.23	6.23	2.42	2.65	0.00	0.55	14.65	14.66
**G10**	0.00	7.27	0.00	1.11	0.00	0.00	0.03	5.30	0.39	0.00	0.00	4.53	18.62	18.68
**G11**	0.76	0.00	0.00	0.30	0.25	0.09	19.93	0.00	0.00	0.00	0.73	0.00	22.04	22.15
**G12**	0.24	7.28	1.44	10.19	0.00	3.56	0.00	0.00	0.00	0.00	0.00	0.30	23.01	23.09

Sample	Gallic acid, mg/100g	Protocatechuic acid, mg/100g	4- hydroxybenzoic acid, mg/100g	Vanilic acid, mg/100g	Chlorogenic acid, mg/100g	Caffeic acid, mg/100g	P-coumaric acid, mg/100g	Rosmarinic acid, mg/100g	Myricetin, mg/100g	Quercitin, mg/100g	Luteolin, mg/100g	Kaempferol, mg/100g	Total poliphenols, mg/100g
**R1**	0.00	0.00	0.00	0.00	0.00	0.04	0.04	0.00	0.00	0.00	0.00	0.00	0.08
**R2**	0.00	0.00	0.00	0.00	0.00	0.09	0.00	0.00	0.00	0.00	0.00	0.00	0.09
**R3**	0.00	0.00	0.00	0.00	0.00	0.05	0.03	0.00	0.00	0.00	0.00	0.00	0.09
**R4**	0.00	0.00	0.00	0.00	0.00	0.00	0.12	0.00	0.00	0.00	0.00	0.00	0.12
**R5**	0.00	0.00	0.00	0.14	0.00	0.00	0.00	0.00	0.00	0.00	0.00	0.00	0.14
**R6**	0.00	0.00	0.14	0.00	0.00	0.00	0.00	0.00	0.00	0.00	0.00	0.00	0.14
**R7**	0.00	0.00	0.00	0.00	0.00	0.15	0.00	0.00	0.00	0.00	0.00	0.00	0.15
**R8**	0.00	0.00	0.00	0.00	0.00	0.10	0.05	0.00	0.00	0.00	0.00	0.00	0.15
**R9**	0.00	0.00	0.00	0.00	0.00	0.15	0.00	0.00	0.00	0.00	0.00	0.00	0.15
**R10**	0.00	0.00	0.00	0.00	0.00	0.00	0.16	0.00	0.00	0.00	0.00	0.00	0.16
**R11**	0.00	0.00	0.00	0.00	0.17	0.00	0.00	0.00	0.00	0.00	0.00	0.00	0.17
**R12**	0.00	0.00	0.00	0.00	0.00	0.11	0.07	0.00	0.00	0.00	0.00	0.00	0.18
**R13**	0.07	0.00	0.00	0.00	0.13	0.00	0.00	0.00	0.00	0.00	0.00	0.00	0.20
**R14**	0.00	0.21	0.00	0.00	0.00	0.00	0.00	0.00	0.00	0.00	0.00	0.00	0.21
**H1**	0.02	0.00	0.00	3.15	0.00	0.00	0.23	5.89	2.34	2.67	0.00	0.55	14.87
**H2**	0.02	0.00	0.00	4.42	0.00	0.37	0.23	5.01	2.11	2.32	0.00	0.41	14.92
**H3**	0.35	13.32	0.00	0.00	0.10	0.06	0.05	0.18	0.33	0.00	0.71	0.00	15.12
**H4**	0.00	6.51	0.99	0.00	1.78	0.47	0.11	0.00	6.60	0.00	0.00	0.00	16.48
**H5**	0.61	2.03	0.65	18.04	1.16	0.07	0.07	0.00	0.00	0.00	0.00	1.18	23.85
**H6**	0.04	0.08	0.00	1.21	0.25	0.00	0.19	3.28	0.14	21.21	0.00	0.26	26.70
**H7**	0.00	0.00	0.00	24.81	0.00	5.53	0.00	0.00	0.00	0.00	0.00	0.00	30.34
**H8**	0.26	0.00	0.00	0.00	0.00	0.20	0.17	51.11	0.00	0.00	0.00	0.00	51.76
**H9**	0.00	0.00	0.00	0.00	0.00	0.00	0.02	60.60	19.75	0.00	0.00	0.00	80.38
**P1**	5.22	0.52	0.00	0.00	0.52	0.10	0.15	0.00	0.00	0.00	0.00	0.00	6.54
**P2**	0.00	0.00	0.00	0.00	0.00	1.84	0.26	4.67	0.00	0.00	0.00	0.00	6.78
**P3**	0.00	0.06	0.00	0.00	0.00	0.00	0.22	4.66	2.14	0.00	0.00	0.00	7.09
**P4**	0.02	0.00	0.00	1.30	0.00	0.00	0.23	5.95	0.00	0.00	0.00	0.00	7.51
**P5**	0.00	0.00	0.00	0.00	0.00	0.00	0.00	0.00	1.93	6.13	0.00	0.00	8.06
**P6**	5.23	0.00	0.00	0.00	0.00	0.01	2.32	0.53	0.00	0.00	0.00	0.00	8.11
**P7**	2.27	0.65	0.49	1.91	0.22	0.12	0.00	2.27	0.00	0.50	0.00	0.00	8.46
**P8**	5.21	0.07	0.52	0.62	1.16	0.07	0.07	0.00	0.00	0.00	0.00	1.21	8.98
**P9**	0.00	0.04	0.00	2.27	0.00	0.00	0.25	5.45	0.00	0.00	1.12	0.00	9.14
**P10**	0.01	0.01	0.00	3.10	0.00	0.08	0.18	5.14	0.36	0.00	0.00	0.41	9.32
**P11**	0.03	0.08	0.00	1.28	0.00	0.00	0.02	4.46	1.09	2.23	0.00	0.16	9.38
**P12**	0.02	0.00	0.00	1.18	0.00	0.00	0.21	5.46	2.26	0.31	0.00	0.44	9.91
**P13**	0.00	0.01	0.00	2.41	0.00	0.00	0.21	4.88	0.00	2.63	0.00	0.56	10.72
**P14**	2.29	0.65	0.28	1.91	0.19	0.09	0.00	5.26	0.00	0.55	0.00	0.00	11.23
**P15**	0.02	0.08	0.00	0.97	0.00	0.00	0.23	5.52	0.12	4.42	0.00	0.60	11.99
**P16**	0.00	0.22	0.00	2.25	0.00	0.00	0.25	6.57	0.14	2.86	0.00	0.48	12.81
**L1**	0.00	0.63	0.00	0.00	0.00	0.28	0.00	0.02	0.00	0.00	0.00	0.00	0.94
**L2**	0.64	0.29	0.00	0.00	0.00	0.03	0.00	0.00	0.00	0.00	0.00	0.00	0.97
**L3**	0.14	0.89	0.00	0.00	0.13	0.00	0.00	0.00	0.00	0.00	0.00	0.00	1.18
**L4**	1.27	0.00	0.00	0.00	0.00	0.00	0.00	0.00	0.00	0.00	0.00	0.00	1.27
**L5**	1.42	0.00	0.00	0.00	0.00	0.00	0.00	0.00	0.00	0.00	0.00	0.00	1.42
**L6**	1.66	0.00	0.00	0.00	0.00	0.00	0.00	0.00	0.00	0.00	0.00	0.00	1.66
**L7**	0.00	0.00	0.00	0.00	0.00	0.03	1.25	0.00	0.41	0.00	0.00	0.00	1.70
**L8**	0.00	0.00	0.00	0.00	0.10	0.04	0.00	0.00	1.59	0.00	0.00	0.00	1.75
**L9**	0.32	0.00	0.00	0.00	0.00	1.20	0.26	0.00	0.00	0.00	0.00	0.00	1.78
**L10**	1.79	0.00	0.00	0.00	0.00	0.00	0.00	0.00	0.00	0.00	0.00	0.00	1.79
**L11**	0.00	0.00	0.00	0.00	0.00	0.49	0.37	0.00	0.94	0.00	0.00	0.00	1.81
**L12**	1.06	0.21	0.50	0.00	0.22	0.00	0.00	0.10	0.00	0.00	0.00	0.00	2.10
**L13**	2.13	0.00	0.00	0.00	0.00	0.00	0.00	0.00	0.00	0.00	0.00	0.00	2.13
**L14**	0.00	0.00	0.00	0.00	0.17	0.09	0.44	0.00	1.31	0.00	0.00	0.20	2.23
**L15**	3.96	0.00	0.00	0.00	0.00	0.00	0.00	0.00	0.00	0.00	0.00	0.00	3.96

**L16**	3.97	0.00	0.00	0.00	0.00	0.00	0.01	0.00	0.00	0.00	0.00	0.00	3.98
**L17**	0.00	0.00	0.30	0.00	0.00	0.00	0.00	4.67	0.00	0.00	0.00	0.00	4.98
**L18**	2.33	0.00	0.00	0.00	0.00	0.00	0.00	0.68	1.92	0.06	0.00	0.00	5.01
**L19**	2.33	0.66	0.16	0.00	0.27	0.04	0.00	1.03	0.32	0.48	0.00	0.00	5.32
**L20**	0.01	1.72	0.50	0.00	2.15	0.00	0.00	1.02	0.00	0.00	0.00	0.00	5.42
**L21**	0.00	0.20	0.54	0.00	0.19	0.00	0.10	4.40	0.00	0.00	0.00	0.00	5.44
**A1**	0.20	0.00	0.00	0.00	0.00	0.00	0.00	0.00	0.00	0.00	0.00	0.00	0.20
**A2**	0.00	0.00	0.02	0.00	0.00	0.00	0.00	0.00	0.00	0.00	0.00	0.00	0.02
**A3**	0.00	0.00	0.00	0.00	0.00	0.00	0.29	0.00	0.00	0.00	0.00	0.00	0.28
**A4**	0.00	0.00	0.00	0.00	0.00	0.04	0.00	0.00	0.00	0.00	0.00	0.00	0.04
**A5**	0.00	0.00	0.00	0.00	0.00	0.00	0.04	0.00	0.00	0.00	0.00	0.00	0.04
**A6**	0.00	0.00	0.00	0.00	0.00	0.04	0.00	0.00	0.00	0.00	0.00	0.00	0.04
**A7**	0.00	0.00	0.00	0.00	0.00	0.05	0.00	0.00	0.00	0.00	0.00	0.00	0.05
**A8**	0.00	0.00	0.00	0.00	0.00	0.05	0.00	0.00	0.00	0.00	0.00	0.00	0.05
**A9**	0.00	0.00	0.00	0.00	0.00	0.05	0.00	0.00	0.00	0.00	0.00	0.00	0.05
**A10**	0.00	0.00	0.00	0.00	0.00	0.05	0.00	0.00	0.00	0.00	0.00	0.00	0.05
**A11**	0.00	0.00	0.00	0.00	0.06	0.00	0.00	0.00	0.00	0.00	0.00	0.00	0.06
**A12**	0.06	0.00	0.00	0.00	0.00	0.00	0.00	0.00	0.00	0.00	0.00	0.00	0.06
**A13**	0.00	0.00	0.00	0.00	0.00	0.06	0.00	0.00	0.00	0.00	0.00	0.00	0.06
**A14**	0.00	0.00	0.00	0.00	0.00	0.06	0.00	0.00	0.00	0.00	0.00	0.00	0.06
**A15**	0.00	0.00	0.00	0.00	0.00	0.04	0.03	0.00	0.00	0.00	0.00	0.00	0.07
**A16**	0.00	0.00	0.00	0.00	0.00	0.06	0.02	0.00	0.00	0.00	0.00	0.00	0.07
**A17**	0.08	0.00	0.00	0.00	0.00	0.00	0.00	0.00	0.00	0.00	0.00	0.00	0.08
**A18**	0.00	0.00	0.00	0.00	0.00	0.08	0.00	0.00	0.00	0.00	0.00	0.00	0.08
**A19**	0.00	0.00	0.00	0.09	0.00	0.00	0.00	0.00	0.00	0.00	0.00	0.00	0.09
**A20**	0.00	0.00	0.00	0.00	0.00	0.09	0.00	0.00	0.00	0.00	0.00	0.00	0.09
**A21**	0.00	0.00	0.00	0.00	0.14	0.00	0.00	0.00	0.00	0.00	0.00	0.00	0.14

Sample	Gallic acid, mg/100g	Protocatechuic acid, mg/100g	4- hydroxybenzoic acid,mg/100g	Vanilic acid, mg/100g	Chlorogenic acid, mg/100g	Caffeic acid, mg/100g	P-coumaric acid, mg/100g	Rosmarinic acid, mg/100g	Myricetin, mg/100g	Quercitin, mg/100g	Luteolin, mg/100g	Kaempferol, mg/100g	Total poliphenols, mg/100g
**G1**	0.01	0.00	0.00	3.16	0.00	0.05	0.23	5.16	2.67	2.46	0.92	0.00	14.67
**G2**	0.00	0.16	0.00	12.76	0.00	0.00	0.27	6.02	0.48	4.26	0.00	0.87	24.81
**G3**	0.00	4.03	4.78	0.00	4.67	1.59	24.23	0.00	0.00	0.00	0.00	0.00	39.30
**G4**	0.00	0.00	0.00	44.81	0.00	0.00	0.00	1.04	0.00	0.00	0.00	0.00	45.85
**G5**	0.00	0.05	0.00	3.00	0.00	0.00	0.22	4.94	2.11	2.20	0.00	0.45	12.97
**G6**	0.04	0.05	0.00	3.10	0.00	0.00	0.24	5.51	2.24	2.32	0.00	0.05	13.54
**G7**	5.23	8.85	0.00	0.00	0.00	0.00	0.00	0.00	0.00	0.00	0.00	0.00	14.08
**G8**	0.02	0.01	0.00	7.25	0.00	0.00	0.02	6.02	0.69	0.00	0.00	0.47	14.47
**G9**	0.02	0.00	0.00	2.55	0.00	0.00	0.23	6.23	2.42	2.65	0.00	0.55	14.65
**G10**	0.00	7.27	0.00	1.11	0.00	0.00	0.03	5.30	0.39	0.00	0.00	4.53	18.62
**G11**	0.76	0.00	0.00	0.30	0.25	0.09	19.93	0.00	0.00	0.00	0.73	0.00	22.04
**G12**	0.24	7.28	1.44	10.19	0.00	3.56	0.00	0.00	0.00	0.00	0.00	0.30	23.01
